# Natural variations in the non-coding region of *ZmNAC080308* contributes maintaining grain yield under drought stress in maize

**DOI:** 10.1186/s12870-021-03072-9

**Published:** 2021-06-30

**Authors:** Nan Wang, Ming Cheng, Yong Chen, Bojuan Liu, Xiaonan Wang, Guojun Li, Yueheng Zhou, Ping Luo, Zhangying Xi, Hongjun Yong, Degui Zhang, Mingshun Li, Xuecai Zhang, Felix San Vicente, Zhuanfang Hao, Xinhai Li

**Affiliations:** 1 Institute of Crop Sciences, Chinese Academy of Agricultural Sciences, Beijing, P.R. China; 2 International Maize and Wheat Improvement Center (CIMMYT), Texcoco, Mexico; 3 College of Agronomy, Hebei Agricultural University, Baoding, P.R. China; 4 State Key Laboratory of North China Crop Improvement and Regulation, Hebei Agricultural University, Baoding, P.R. China; 5 College of Agronomy, Henan Agricultural University, Zhengzhou, P.R. China

**Keywords:** Maize (*Zea Mays* L.), NAC transcription factor, Natural variations, Non-coding region, Drought tolerance

## Abstract

**Background:**

Natural variations derived from both evolutionary selection and genetic recombination, presume to have important functions to respond to various abiotic stresses, which could be used to improve drought tolerance via genomic selection.

**Results:**

In the present study, the NAC-encoding gene of *ZmNAC080308* was cloned and sequenced in 199 inbred lines in maize. Phylogenetic analysis showed that *ZmNAC080308* is closely clusteredinto the same group with other well-known NAC genes responding to improve drought tolerance. In total, 86 SNPs and 47 InDels were identified in the generic region of *ZmNAC080308*, 19 of these variations were associated with GY (grain yield) in different environments. Nine variations in the 5’-UTR region of *ZmNAC080308* are closely linked, they might regulate the gene expression and respond to improve GY under drought condition via *Sp1*-mediated transactivation. Two haplotypes (Hap1 and Hap2) identified in the, 5’-UTR region using the nine variations, and Hap2 containing insertion variants, exhibited 15.47 % higher GY under drought stress condition. Further, a functional marker was developed to predict the drought stress tolerance in a US maize inbred line panel. Lines carrying Hap2 exhibited > 10 % higher GY than those carrying Hap1 under drought stress condition. In *Arabidopsis*, overexpression *ZmNAC080308* enhanced drought tolerance.

**Conclusions:**

*ZmNAC080308* is an important gene responding to drought tolerance, a functional marker is developed for improving maize drought tolerance by selecting this gene.

**Supplementary Information:**

The online version contains supplementary material available at 10.1186/s12870-021-03072-9.

## Background

Food security in terms of grain yield will become more difficult to achieve with limited arable land as human populations grow. Crop yields are suffered from different abiotic stresses and it will continue to be challenged due to climate changes. The latest report from FAO indicates billions of US dollars were lost in agricultur from 2005 to 2015 due to environmental disasters. More than 30 % of these losses were caused by drought, which alone resulted in 29 billion US dollars in agricultural losses (http://www.fao.org/news/story/en/item/1106977/icode/). Therefore, improveing the crop tolerance to abiotic stresses, especially drought, becomes very urgent.

Transcription factors (TFs) play important roles in molecular mechanisms of stress tolerance in plants [1]. In particular, NAC is a member of a large plant-specific family of TFs that includes NAM, ATAF, and CUC with important diverse functions. The first NAC gene, i.e. NAM (no apical meristem), was identified in petunia [2]. Subsequently, CUC1 and CUC2 were identified in *Arabidopsis*,because the N-terminus of CUC2 was highly conserved with those of NAM and ATAF, this N-terminal region was then named the NAC domain [3]. As shown in the TF database (http://planttfdb.cbi.pku.edu.cn/), more than 500 NAC genes have been identified in *Arabidopsis*, rice, and maize, and these genes have been categorized into different subgroups [4–6]. Therefore, NACs involved in the plant stress-responsive subgroups are known as SNACs (stress-responsive NACs) [7–10]. These SNACs are involved in many stress-responsive metabolic pathways in plants including ROS generation, hormone function, osmotic adjustment, Ca^2+^ signaling, secondary metabolic processes, etc. [11], where activated NACs can regulate the expression of downstream elements.

Recently, researchers have focused more on the function of NACs in abiotic stress responses, especially in crops. So far, most of the drought-related NACs reported in major crops are positive regulators. Overexpression of *ZmNAC55* in *Arabidopsis* can enhance drought tolerance compared to wild-type lines [12]. *TaNAC2* is a NAC TF in wheat that could enhance resistance to drought, salt, and freezing stresses in *TaNAC2*-transgenic *Arabidopsis* [13]. The root-specific expressed rice gene *OsNAC10* can improve resistance to abiotic stress when it is overexpressed. Moreover, overexpression *OsNAC10* in rice increase 25–42 % GY under drought stress conditions in the field [14]. Several other NAC TFs, such as *SNAC1* [15], *ONAC045* [16], *SNAC2* [17], *OsNAC5* [18], *OsNAC6* [19], *OsNAP* [20], *ZmNAC111* [21], and *TaNAC29* [22], have also been identified, these genes have important functions responding to abiotic stresses tolerance, which are useful for developing stress-tolerance crops.

Identification natural variations in genes associated with target traits becomes more important, especially for quantitative traits including drought tolerance, as some *cis*- or *trans*-regulatory functions involved in responses to abiotic stress tolerance can be lost or gained, due to natural variations in these genes [23]. In maize, many natural variants associated with drought tolerance have been identified. Two InDel variations in the upstream region of *ZmVPP1* were identified, which were significantly associated with drought resistance in maize. The haplotype of the insertion variants containing three MYB-binding domains, has greater drought tolerance than the haplotype carrying deletion variants [24]. Another natural variant in the 5’-UTR of *ZmPP2C-A10* deletes an ERSE (endoplasmic reticulum stress response element), the loss of this variant prohibits the expression of *ZmPP2C-A10* during ER stress. Interestingly, disabling the *ZmPP2C-A10* expression results in drought tolerance improvement in maize [25]. Moreover, some natural DREB (dehydration responsive element binding protein) variants in maize have also been reported, they are significantly associated with drought stress tolerance, which is also useful for improving drought tolerance in maize [26].

So far, natural variations associated with drought tolerance have only been reported in *ZmNAC111* [21], variants in other NAC genes associated with drought tolerance have not been discovered. In the present study, we used maize RNA-Seq data to identify a gene encoding a NAC TF, which has relatively high homologies to other known drought response-related NACs. Association analysis revealed that the variants in *ZmNAC080308* are significantly associated with GY in maize, especially under drought-stress conditions. Haplotype of *ZmNAC080308 variants* associated with drought stress tolerance were identified, A functional marker was developed and validated in an association mapping panel, which can be used for improving drought stress tolerance in maize.

## Results

### ZmNAC080308 is a nuclear-localized TF in the abiotic stress-responsive SNAC TF subfamily

**Fig. 1:**
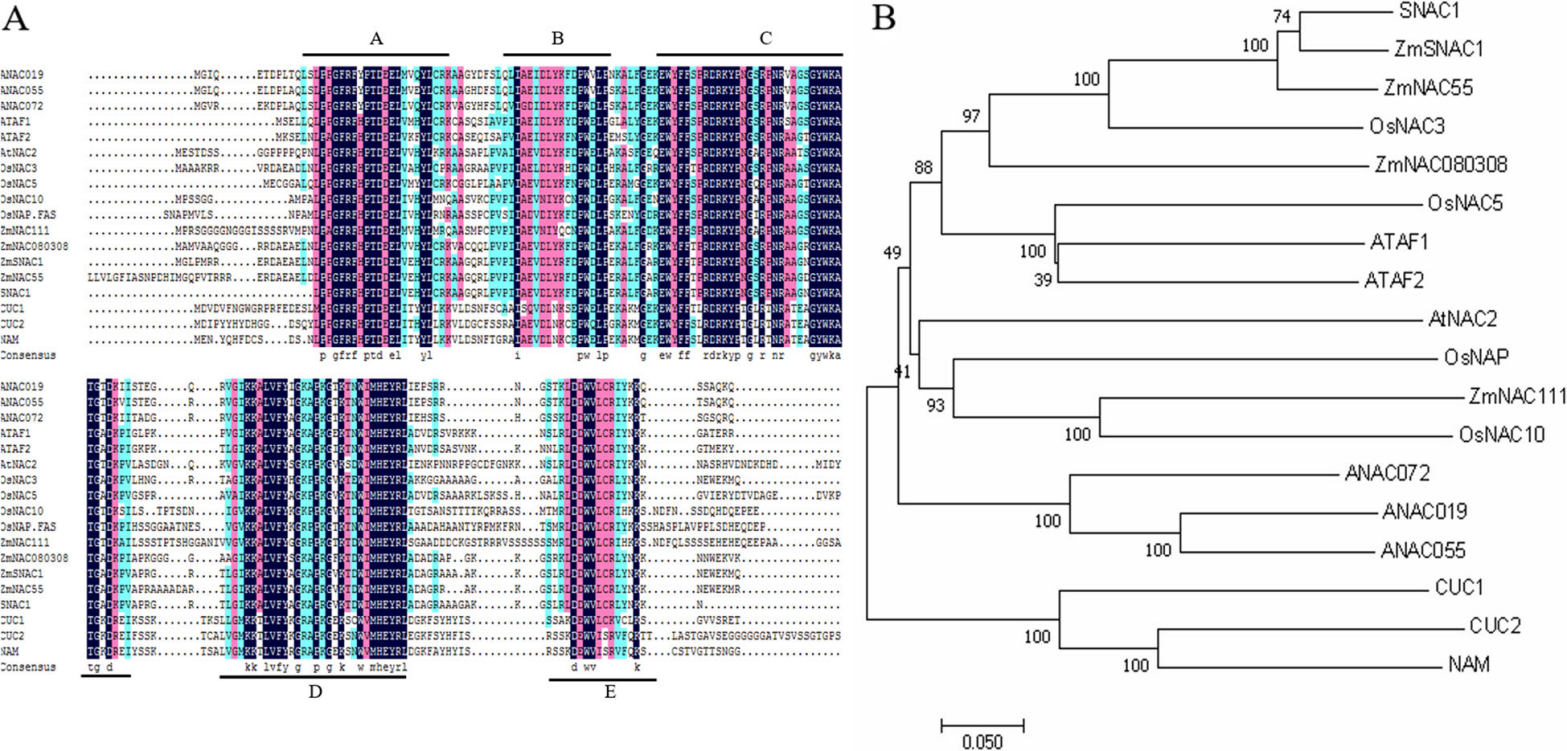
Amino acid sequence comparison and phylogeny of *ZmNAC080308* and other known NAC proteins in maize, rice, and *Arabidopsis*. (**A**) NAC domain sequence alignment, with identical amino acids in dark blue and similar amino acids in pink or light blue. Five highly conserved regions were identified (**A**-**E**). (**B**) Evolutionary relationships between ZmNAC080308 and other NACs. The optimal tree with the sum of branch lengths = 3.94203309 is shown. The percentages of replicate trees in which the associated taxa clustered together in the bootstrap with 1000 replicates are shown next to the branches

As compared with other NAC TFs, *ZmNAC080308* contained the conserved NAC domain including five complete consensus NAC subdomains (A, B, C, D, E) (Fig. 1 A). Phylogenetic analysis showed that genes with similar functions tended to cluster together, the stress-responsive SNAC TFs clustered into one branch with ATAF, while NAM and CUC, which were reported involving in regulation the plant growth and development, appeared in the other branches (Fig. 1B). *ZmNAC080308* was more closely related to *OsNAC3*, *ZmNAC55*, *ZmSNAC1*, and *SNAC1*, according to our phylogenetic analysis. These four genes are associated with drought stress responses in plants [5, 12, 15, 27], indicating that *ZmNAC080308* might also be involved in drought stress responses in maize.

**Fig. 2:**
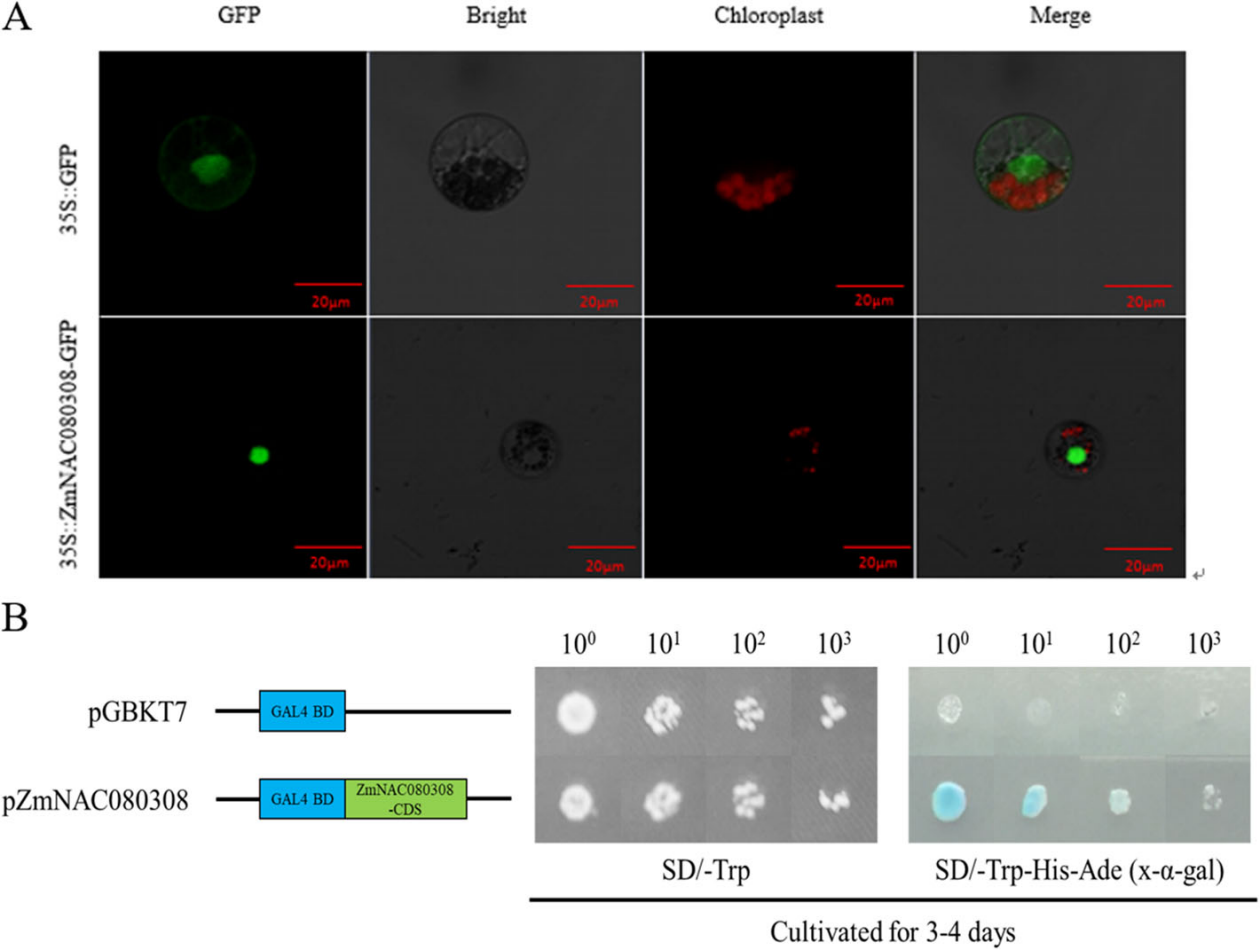
Subcellular localization (**A**) and transactivation assay (**B**) of *ZmNAC080308*. The transformed strains were diluted 1:10^0^, 1:10^1^, 1:10^2^, 1:10^3^, and plated separately on SD/-Trp and SD/-Trp/-His (x-gal) medium

We subsequently transformed *ZmNAC080308*-GFP into maize protoplasts and detected strong GFP signals in the nuclei of the transformed cells, indicating nuclear localization of the ZmNAC080308-GFP fusion protein (Fig. 2 A). Next, we used two-hybrid transactivation assays to investigate whether the ZmNAC080308 protein could activate the expression of reporter genes AH109. The full-length CDS of *ZmNAC080308* was cloned into the pGBKT7 vector, then the constructed or the empty vector was transformed into yeast strain AH109, respectively. As shown in Fig. 2B, both transformants grew well on the SD/-Trp medium, but only the strain containing pGBKT7-*ZmNAC030803* grew on the SD/-Trp/-His medium and showed galactosidase activity. Therefore, *ZmNAC080308* transcription factor was a nuclear-localized protein with transcriptional activity in maize.

### Expression of ***ZmNAC080308*** in tassel and seedling

Firstly, gene expression in young tassels harvested from ten inbred lines under well-watered (WW) and drought-stressed (DS) conditions were analyzed using RNA-Seq data (Wang et al., 2018). Interestingly, results showed that the transcript expression of *ZmNAC080308* under DS conditions differed significantly from that under WW conditions in five maize lines (Tie7922, X178, Dan340, Ji81162 and CA339) at the thresholds of false discovery rate (FDR) ≤ 0.001, and an absolute value of the Log_2_Ratio ≥ 1. In the inbred lines of Tie7922, X178, and Dan340 showing better drought tolerance, the average abundance of *ZmNAC080308* transcripts increased 2.14-fold after drought treatment, while it decreased 1.68-fold after drought treatment in inbred lines of Ji81162 and CA339 (Table 1), showing that the differences in transcript expression could be guided by the signals of drought stress.

**Table 1:** Expression of *ZmNAC080308* in young tassels of five inbred lines responding differently in drought tolerance under well-watered (WW) and drought-stressed (DS) conditions

Line	Drought Tolerance^a^	WW Control-RPKM	DS Drought-RPKM	Ratio^b^	Up-Down	P-value	FDR
Ji81162	HS	2.65	1.23	-1.11	Down	4.14E-09	1.60E-08
CA339	S	0.25	0.05	-2.24	Down	4.07E-03	1.19E-02
Dan340	M	0.65	5.39	3.06	Up	1.95E-88	8.92E-87
X178	R	3.25	9.20	1.50	Up	5.64E-51	5.13E-50
Tie7922	HR	0.49	1.74	1.84	Up	1.11E-10	6.97E-10

^b^. Evaluation of the drought tolerance of the five lines was based on Hao et al., 2011. Highly drought resistance *HR* drought resistance; *R* middle drought resistance; *M* drought susceptible (S) and highly drought-susceptible (HS)

^b^. The ratio was calculated following the formula: Ratio = log2(Drought-RPKM/ Control-RPKM)

**Fig. 3:**
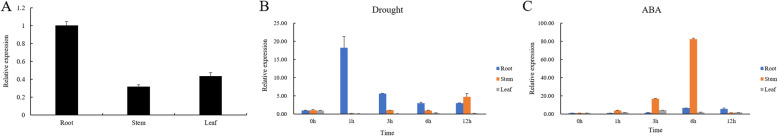
Relative expression of *ZmNAC080308* in roots, stems, and leaves of seedling (**A**) after drought (**B**) and ABA (**C**) treatment

qRT-PCR (quantitative Reverse-Transcription Polymerase Chain Reaction) was used to analyze the relationship between *ZmNAC080308* expression and drought stress tolerance in maize seedlings. *ZmNAC080308* highly expressed in root and the lower portions of the stem (Fig. 3 A). After drought stress treatment, the transcript expression of *ZmNAC080308* increased significantly in root, a peak was identified at 1 h after stress treatment. However, after exogenous ABA treatment, *ZmNAC080308* transcript expression increased by more than 80-fold in stem (Fig. 3B and C). This result presumes that *ZmNAC080308* has a function regulating abiotic stresses in ABA pathway.

### Genetic characterization of ***ZmNAC080308***

We then sequenced the *ZmNAC080308* gene in 199 NLs. A total of 3065 bp of sequence data was obtained from each line, including three exons, two introns, a 645-bp untranslated region and a 1232-bp promoter sequence (Table 2). After aligning the sequence of *ZmNAC080308*, 86 SNPs and 47 InDels were identified among these 199 inbred lines. Among all these SNPs and InDels, 72 variations were identified in the promoter region. Except for the promoter and the 5’-UTR regions, the exon III contained the highest SNP density, with an average of one SNP per 15 bp, while the intron I contained the most InDel density with an average of one InDel per 26 bp. No InDels were identified in the coding region. Among the 32 SNPs identified in exons, eight SNPs were non-synonymous mutations, and the other 24 SNPs were synonymous mutations. Average minor allele frequency (MAF) value was 0.22 L across all the 133 variations, with the lowest average MAF value of 0.28 in the promoter region and the highest average MAF value of 0.50 in the Exon I region.

**Table 2:** Summary information of nucleotide polymorphisms in *ZmNAC080308*

	Promoter	5’-UTR	Exon I	Intron I	Exon II	Intron II	Exon III	3’ UTR
Length (bp)	1232	386	199	158	284	103	444	259
Number of SNPs	36	10	1	7	1	1	29	1
Number of InDels	36	4	0	6	0	1	0	0
MAF	0.28	0.26	0.07	0.07	0.15	0.12	0.15	0.10

**Fig. 4:**
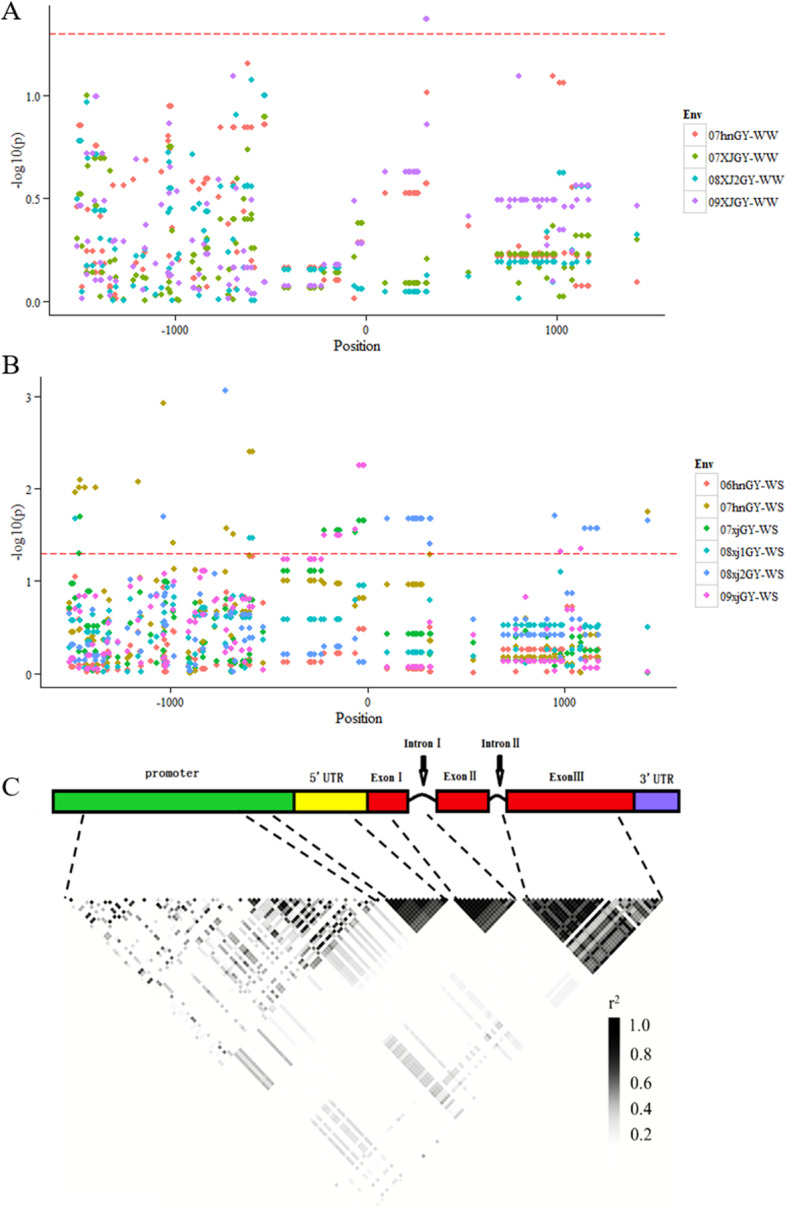
*ZmNAC080308*-based association mapping and linkage disequilibrium analysis. Association analysis of gene sequence variation with maize GY under well-watered (WW) (**A**) and drought-stressed (DS) (**B**) conditions. (**C**) Pairwise LD of polymorphisms in *ZmNAC080308*

Linkage disequilibrium (LD) analysis was performed on all the 133 variants using Haploview. Three LD blocks (r^2^ > 0.6) were identified in the 5’-UTR region, part of Exon I and Intron I region, and the Exon III, respectively (Fig. 4 C). Several variants in the promoter region exhibited strong LD, but most of other variants were not tightly linked with each other.

### Variations in the `***ZmNAC080308*** gene are associated with GY under WW and DS conditions in maize

A candidate gene association study was used to detect the variants in *ZmNAC080308* that were significantly associated with drought stress tolerance in maize. Firstly, the association analysis between the variants and GY was performed, considering the importance of GY in evaluating drought stress tolerance. Using the Mixed Linear Model (MLM) method, 64 variations were detected that were significantly associated with GY in all ten environments. Among these 64 variations, only four variations in Intron I region were identified under WW condition, the rest of the 60 variations were identified only under DS conditions (Fig. 4A and B). Seven variations had *p* values less than 0.01, including four markers in the promoter region and three markers in the 5’-UTR region, each marker explained an average of 6.10 % of the variation of GY.

A total of 19 variations including five InDels and 14 SNPs were detected that were significantly associated with GY in at least two environments (*p* < 0.05) (Fig. 4A and B), each variation explained an average of 4.45 % of the phenotypic variation of GY. All of the variations significantly associated with GY werein the non-coding regions of the *ZmNAC080308* gene (five in the promoter, nine in the 5’ UTR, four in Intron, and one in the 3’ UTR). Strong LD (r^2^ > 0.8) was observed among the variations located in the 5’-UTR, InDel-600, and SNP-586 in the promoter region, as well as between the four variations within the Intron I region (Fig. 4 C).

### Variations in the 5’-UTR of ***ZmNAC080308*** significantly associated with GY under the drought stress condition

**Fig. 5:**
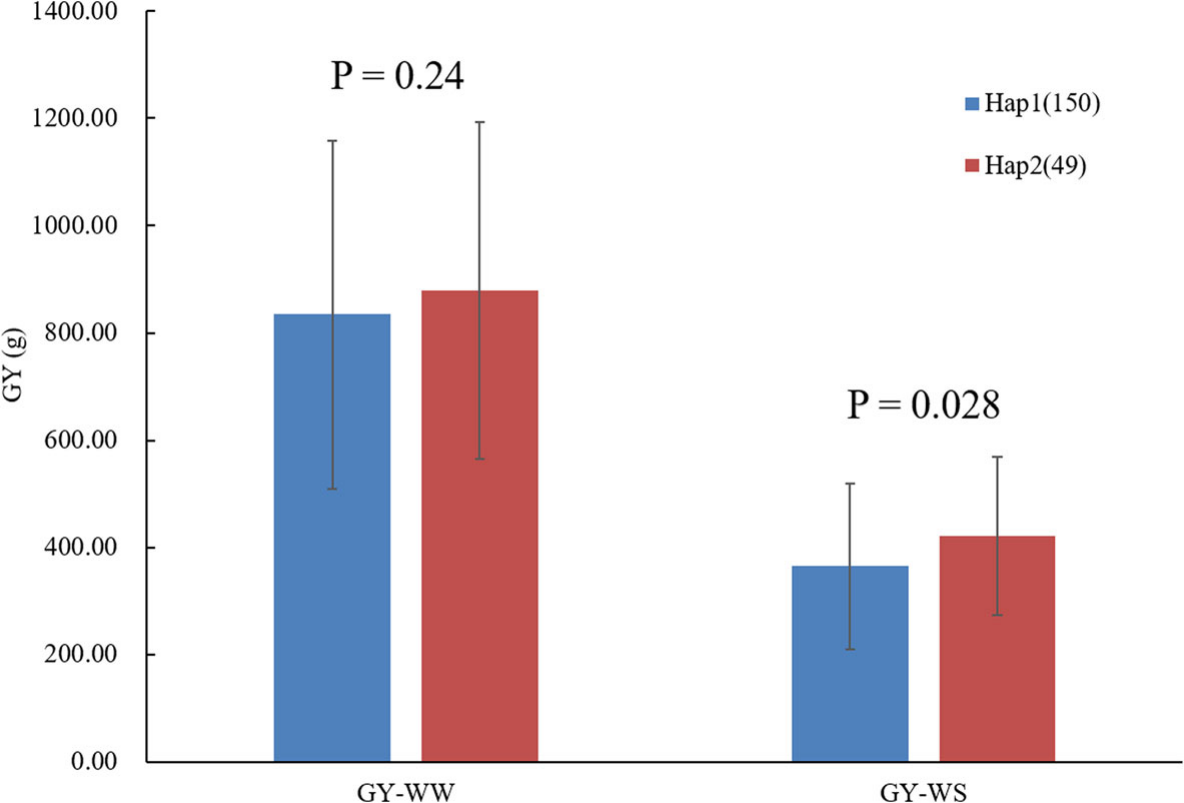
Phenotypic comparisons of two *ZmNAC080308* haplotypes in Chinese inbred lines (NLs)

*ZmNAC080308* was significantly associated with GY, especially under DS conditions, and the variations in the non-coding regions of the gene play an important role in regulating GY under drought stress conditions in maize. Among all the variations in *ZmNAC080308* associated with GY under DS, nine variations in the 5’-UTR, including seven SNPs and two InDels, were in high LD (Table 3; Fig. 4). The 5’-UTR was important in regulating the gene expression, all of the inbred lines were divided into two haplotypes built with the nine variations in the 5’-UTR of *ZmNAC080308*. The two haplotypes had similar GY values under WW conditions, the GY of Hap2 was 421.17 g/plot under DS conditions, which was 15.47 % higher than that of Hap1 under the same conditions (*p* = 0.028) (Fig. 5). Then the *cis*-acting elements in the 5’-UTRs of the two haplotypes were analyzed to identify the differences in these elements between the variants. As expected, several *cis*-acting elements related to stress responses were present in the 5’-UTR region of *ZmNAC080308* (Supplemental Fig. S1), including an ABRE (ABA-responsive element), a DRE (dehydration-responsive element), a CGTCA sequence motif (involved in MeJA response), and a G-box (involved in light response) (Supplemental Fig. S1A). The presence of these motifs suggested that *ZmNAC080308* might be regulated during drought stress responses. Moreover, an additional *cis*-acting element, *Sp1* (light-responsive element), was caused by a nucleotide change from T to C at 42 bp upstream of the start codon of *ZmNAC080308*, which was identified in Hap2 genotypes (Supplemental Fig. S1B). Interestingly, relative luciferase activity was significantly higher under control of the 5’ -UTR from Hap2 than that under the 5’-UTR from Hap1 in both controlled (*p* = 1.69E-07) and ABA-treated (*p* = 2.99E-06) conditions (Supplemental Fig. S1C). These results demonstrated that the mutations in the 5’-UTR of *ZmNAC080308* might functionally regulate the gene expression between these two haplotypes.

**Table 3:** Genotypic information of the two haplotypes (Hap 1, Hap2) of *ZmNAC080308*

Marker No.	77	78	79	80	81	82	83	84	85
Position (bp)	-220	-219	-155	-147	-142	-60	-42	-23	-20
Hap1 (150)	T	T	T	---	--	C	T	C	C
Hap2 (49)	A	C	G	GGC	GT	T	C	G	G

150 and 49 are the number of genotypes

### Development of functional marker for prediction and selection of drought tolerant lines

**Fig. 6:**
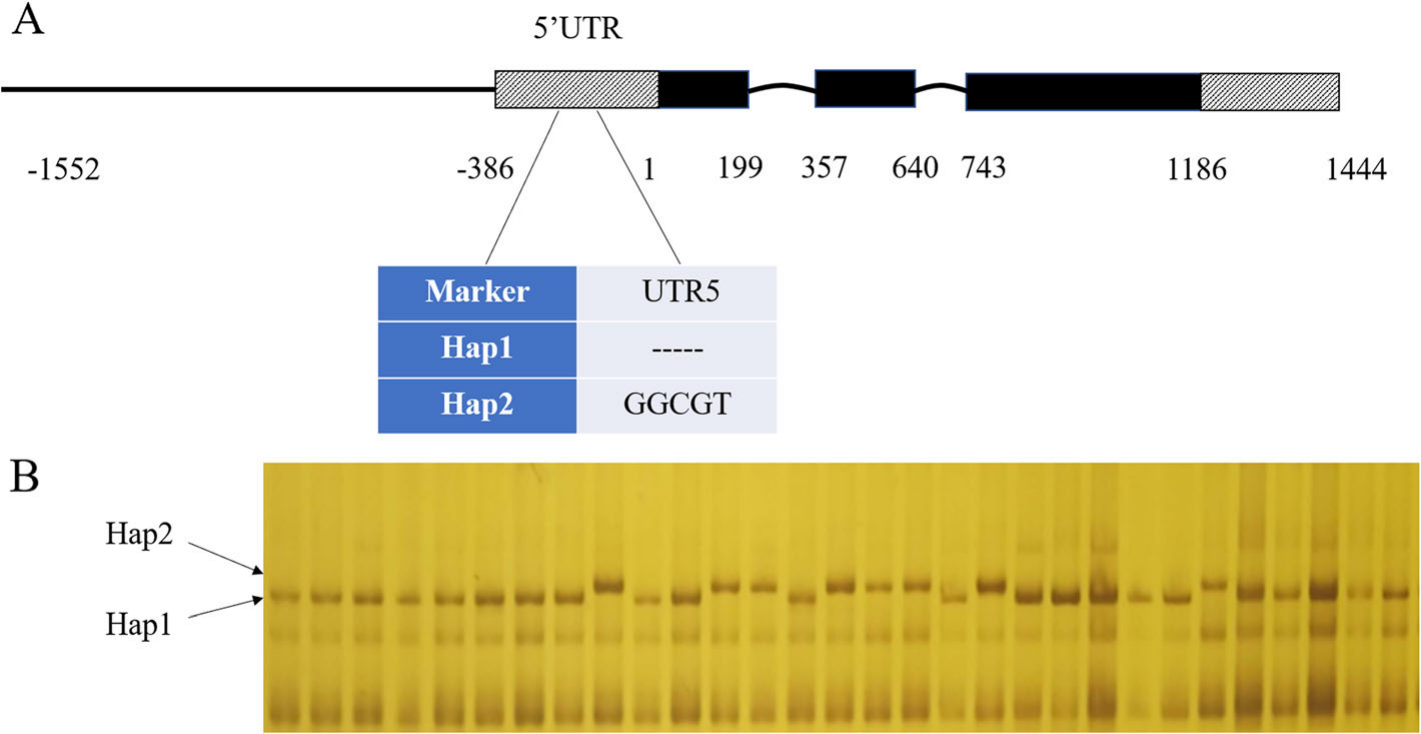
Development of functional marker for ZmNAC080308. (**A**) The functional marker was developed based on the two InDels (Marker 80 and Marker 81) in the 5’ UTR of ZmNAC080308. (**B**) Gel photo of the off-PVP US maize inbred lines screened with the functional marker (Hap1, and Hap2) on SDS-PAGE

To distinguish the two haplotypes of *ZmNAC080308*, a functional marker was developed based on the two InDel variations in the 5’-UTR region. This marker was then tested in another maize inbred line panel containing 187 US off-PVP inbred lines. These inbred lines were then classified into two haplotypes by screening all genotypes on SDS-PAGE (Fig. 6). In total, Hap1 existed in 116 inbred lines carrying the deletion variation, Hap2 existed in 70 inbred lines carrying the insertion variation. Hap2 had an average GY value of 63.22 g/plot under WS condition, which was higher than that of Hap1 under the same condition, but the differences were not significant (*p* > 0.05). However,, the average GY of Hap2 was 11.30 % significantly higher than that of Hap1 (*p* = 0.03) under DS conditions (Table 4). These results were consistent with those of the previous experiment, which proved that the variations in the 5’-UTR of *ZmNAC080308* were related to maize drought resistance. The functional marker developed by the present study could be used to select the drought stress tolerance inbred lines.

**Table 4:** GY performance of the two haplotypes in ALs under well-watered (WW) and drought-stressed (DS) conditions, 116 ALs carrying Hap1, and 70 lines carrying Hap2

Env.	Treatment	Hap1(116)	Hap2(70)	Hap2-Hap1	p-value
Hainan	WW	902.89	1027.18	124.29	0.01**
	DS	439.22	514.04	74.81	0.01**
Xinjiang	WW	1434.35	1417.50	-16.85	n.s.
	DS	547.98	583.71	35.73	n.s.
Average	WW	1168.62	1231.84	63.22	n.s.
	DS	490.23	545.64	55.41	0.03*

### Overexpression of ***ZmNAC080308*** enhances drought resistance in transgenic ***Arabidopsis***

**Fig. 7:**
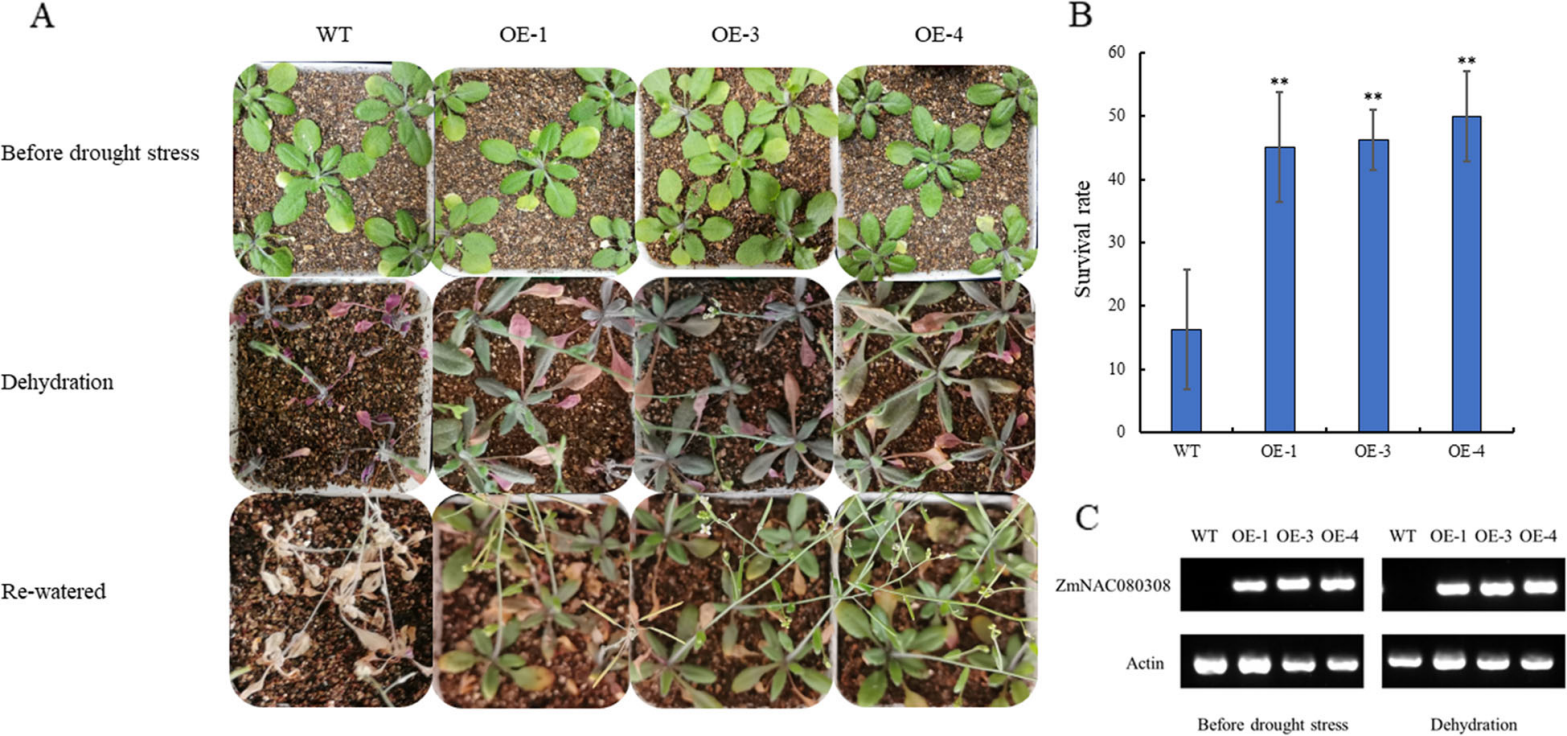
Phenotype of the 35 S:ZmNAC080308 transgenic *Arabidopsis*. (**A**) Drought tolerance of transgenic Arabidopsis plants overexpressing ZmNAC080308. Wild-type (WT) and OE-1, OE-3 and OE-4 transgenic plants are shown. (**B**) Statistical analysis of survival rates after the drought-stress treatment. The average survival rates and standard errors were calculated based on data obtained from three independent experiments. Significant differences were determined by a t-test. **P < 0.01. (**C**) Expression of ZmNAC080308 transcripts in the transgenic *Arabidopsis*

Three independent transgenic lines (OE1, OE3, and OE4) of *ZmNAC080308* overexpression in *Arabidopsis* were chosen for further analysis. During the process of drought stress, it was observed that most wild-type (WT) plants withered at earlier stage, while the *35 S::ZmNAC080308* transgenic plants remained in green condition (Fig. 7 A). After withholding water for 3 weeks, most leaves of both WT plants and the transgenic plants began to dry for rehydration. Further statistical analysis of the survival rate of the *Arabidopsis* after drought stress revealed that ~ 50 % of transgenic plants were still alive, whereas ~ 85 % of WT plants died (Fig. 7B). These results showed that overexpression of *ZmNAC080308* can improve drought tolerance of the transgenic *Arabidopsis*.

## Discussion

### ***ZmNAC080308*** encodes a NAC transcription factor affecting drought stress tolerance in maize

NAC TFs and their SNAC subfamily comprise an important group of genes involved in plant abiotic stress responses. Previous phylogenetic analysis showed that, 16 NAC genes belong to the SNAC subfamily in maize [6]. Among these 16 SNAC genes, three NAC genes including *ZmNAC010312* (*GRMZM2G347043*, *ZmSNAC1*) [27], *ZmNAC050436* (*GRMZM2G336533*, *ZmNAC55*) [12], and *ZmNAC100475* (*GRMZM2G127379*, *ZmNAC111*) [21] have also been reported responding to drought stress tolerance in maize. In the present study, we cloned another maize SNAC subfamily gene of *ZmNAC080308* based on RNA-Seq data analysis, this gene presumed to respond to drought stress resistance. After DS treatment, the transcript abundance of *ZmNAC080308* was higher in the young maize tassels of inbred lines showing moderate to high drought stress,, while it was relatively low in the susceptible inbred lines (Table 1). The 5’-UTR of *ZmNAC080308* was analyzed, the stress responsive *cis*-elements, such as ABRE and DRE, were identified (Supplemental Fig. S1). Further, just like that of other SNAC genes such as *ZmNAC55* [12], *OsNAC3* [5], and *ZmSNAC1* [27]. qRT-PCR analysis also showed that expression of *ZmNAC080308* could be induced by both ABA and drought stresses in roots and stems (Fig. 3), However, the expression of *ZmNAC080308* under drought and ABA stresses differed between roots and stems. In roots, the expression of *ZmNAC080308* was more sensitive to drought, whereas the expression of *ZmNAC080308* rose more rapidly in stems after ABA treatment (Fig. 3). Results show that the expression of the *ZmNAC080308* gene presumed responding to drought stress through both ABA-dependent pathway in stem and ABA-independent pathway in root, which was consistent with the results of our previous study, the *ZmNAC080308* gene contains both ABRE and DRE in its promoter [6]. Overexpression of *ZmNAC080308* enhances drought tolerance in transgenic *Arabidopsis*. The survival rate of the transgenic *Arabidopsis* was not as high as expected, which might cause by the severe drought stress, although it was still much higher than the survival rate of WT. All the results in the present study indicate that *ZmNAC080308* variants play an important role responding to drought stress through both ABA-dependent and ABA-independent pathways in maize.

A total of 32 SNPs in the *ZmNAC080308* coding region were identified, eight of them were non-synonymous mutations, which located in Exon III region. Association analysis showed that two of the eight variants were significantly associated with GY evaluated under DS conditions. However, the effects of both variants were only identified in one of five tested environments (location × year) i.e. SNP-118 was detected in 2008 in Xinjiang and SNP120 was detected in 2009 in Xinjiang, this result indicates that the effects of these two associations were not stable. Moreover, TFs can be involved in multiple pathways, the variants identified in the present study might also be related to other traits, such as flowering time. Therefore, further analyses should be conducted to detect the associations between *ZmNAC080308* and other important agronomic traits.

### Variations in the non-coding regions of ***ZmNAC080308*** affecting the drought stress tolerance

Candidate gene-based association analysis was used to detect the associations between genetic polymorphisms and the target traits. Compared to genome-wide association (GWAS) strategies, the candidate gene approach requires fewer genetic markers, it can minimize the statistical issues caused by multiple-testing across the entire genome [28]. Candidate gene association analysis was firstly used to analyze the association between the *dwarf8* gene and flowering time in maize [29], where nine flowering time-associated polymorphisms were identified. In a previous study, association analysis showed that a deletion variant in the 5’-UTR of *ZmPP2C-A10* decreased the gene expressionlevel, which resulted in maize drought tolerance improvement [25]. Another drought tolerance-related gene, *ZmVPP1*, was also identified by GWAS and candidate gene association analysis, two InDel variants on the upstream of its start codon and two variants within its CDS were detected, which were significantly associated with drought tolerance in maize. Furthermore, the drought tolerance-related haplotype of *ZmVPP1* was identified [24]. An insertion variant in the promoter region of *ZmNAC111* was also associated with drought tolerance in maize. This variant correlated with lower expression of the *ZmNAC111* gene, which affected DNA and histone methylation and caused maize seedlings to be more sensitive to drought stress [21]. In the present study, nine highly linked polymorphisms including two InDel variants in the 5’-UTR of *ZmNAC080308* were identified and associated with GY under drought stress conditions. Two haplotypes of *ZmNAC080308* were built using these variations, Hap2 exhibited higher GY than Hap1 under drought stress conditions. In addition, a functional marker in the 5’-UTR of *ZmNAC080308* (Fig. 6) was developed based on the InDel variants and an off-PVP maize inbred line panel was genotyped with this functional marker. Results showed that the GY of the inbred lines carrying the favorable genotypes of this functional marker was 11.30 % higher, indicateing that this functional marker could be used in MAS for improving droughttolerance in temperate maize.

The 5’-UTR is an important region that regulates the expression of eukaryotic genes (Barrett et al., 2012; Cenik et al., 2011). This study detected a SNP located at 42 bp upstream of the start codon of *ZmNAC080308*, which creates the *cis*-acting element *Sp1* in Hap2 genotypes (Supplemental Fig. S1B). A previous study showed that promoters from *Sp1* mutant lines had reduced GUS expression compared with promoters isolated from wild-type lines [30]. Moreover, the *Sp1* cis-element is one of the cis-elements that are presented in the promoter region of expressed drought responsive genes [31]. Therefore, we hypothesized that this kind of mutation in *ZmNAC080308* could modulate drought-stress tolerance in maize. Further, the 5’-UTR of the *ZmNAC080308* Hap2 genotype showed higher transcriptional activity than that of the Hap1 genotype in the dual-luciferase reporter assay system (Supplemental Fig. S1D). Therefore, we tested the expression level of the *ZmNAC080308* gene in different haplotypes using RNA-Seq analysis. Among the five lines we tested, only X178 carries Hap2, the other four lines carry Hap1 (Table 1). The RPKM (Reads Per Kilobase per Million mapped reads) value of *ZmNAC080308* in X178 was higher than that of the other four lines under both WW and DS conditions, indicating that the addition of the *Sp1* motif can increase the expression of *ZmNAC080308*, which is related with drought stress tolerance in maize. Finally, the drought tolerance ability of the *35 S::ZmNAC080308* were tested in transgenic *Arabidopsis*. The survival rate of the transgenic plants was much higher than that of the WT plants (Fig. 7). These results confirm that *ZmNAC080308* functions as a positive regulator of drought stress, it can be used for maintaining the GY under drought stress conditions in maize.

## Conclusions

*ZmNAC080308* is an important gene responding to drought tolerance and its marker from variations could be used as a functional identifier for improving maize GY under drought stress.

## Methods

### Collection of GY data under well-watered (WW) and drought-stressed (DS) conditions

In the present study, GY data for 389 lines in two association panels were collected and analyzed. One association panel included 199 inbred lines commonly used in China (NLs). The GY data for the NLs grown under WW conditions were collected in Sanya, Hainan Province (18°14′ N, 109°31′ E) in 2007, and in Urumqi, Xinjiang Province (43°54′ N, 87°28′ E) in 2007, 2008, and 2009. The GY data for the NLs grown under DS conditions were collected in Sanya, Hainan Province (18°14′ N, 109°31′ E) in 2006 and 2007, and in Urumqi, Xinjiang Province (43°54′ N, 87°28′ E) in 2007, twice in 2008 and once in 2009. The drought-stress conditions were artificially imposed by withholding irrigation around 20 d before maize flowering depending on the weather condition. More detailed information regarding these data can be found in our previous studies [32, 33].

The other association panel was composed of 186 US off-PVP inbred lines (ALs; US lines no longer under patent). This panel was planted in a α-lattice design in Hainan and Xinjiang, the same locations as the NLs in 2013 with two watering treatments (WW and DS conditions). The experiment design and irrigation methods were the same as those used for the NLs, which have been described in our previous study [32]. In brief, each line was planted two replications in single-row 4-m long plot with 20 cm between plants, and 21 plants per row. The GY data of ALs under each treatment was collected three weeks after harvest.

### Patterns of mRNA expression of ***ZmNAC080308***

The expression levels of *ZmNAC080308* in young tassels under normal and drought-stressed conditions were from a previous RNA-Seq study [34]. Expression data of five inbred lines including Ji81162, CA339, Dan340, X178, and Tie7922, exhibited different degrees of drought stress tolerance (Table 1).

In the present study, qRT-PCR was conducted to characterize the expression of *ZmNAC080308* after drought or ABA treatment at seedling stages. Seeds of inbred line Tie7922 were first planted in quartz sand in the greenhouse and watered every other day. Endosperms were removed at the V1 stage, and then seedlings of similar sizes were transferred into buckets filled with Hoagland’s nutrient solution for continued growth. The solution was refreshed every 2 d until the V2 stage. The seedlings were then transferred into Hoagland’s solution containing 20 % PEG-6000 for the drought treatment or 100 µM ABA for the ABA treatment. The roots, leaves, and stems of each treated material and controls were sampled separately after treatment at 0 h, 1 h, 3 h, 6 h, and 12 h. Five samples from each treatment at the same sample collection were mixed equally as a replication, and two replications were used in this study.

Total RNA was isolated from samples using TransZol Up (Transgen, China) according to the manufacturer’s instructions. A FastQuant RT Kit (Tiangen, China) was used to reverse transcribe the first-strand cDNA from 1000 ng of total RNA. qRT-PCR was then performed using a SuperReal PreMix Plus Kit (SYBR Green) (Tiangen, China) with IQ5 (Bio-Rad, US). The relative expression level of *ZmNAC080308* was calculated relative to the expression level of the maize *NADPH* gene using the 2^−ΔΔCt^ method [35].

### Sequencing of full-length ***ZmNAC080308*** in NLs

The seeds of all NLs were planted in three 72-cell germination boxes in the greenhouse. One leaf was sampled from each line at the V2 stage, and genomic DNA was extracted from each line using the CTAB method. The gene sequence of *ZmNAC080308* was downloaded from the National Center for Biotechnology Information database (NCBI, https://www.ncbi.nlm.nih.gov/). The gene structure of *ZmNAC080308* was predicted using the online tool FGENESH (http://www.softberry.com) and its 5’-UTR was searched for *cis*-acting regulatory elements using the PlantCARE database [36]. We then cloned the full-length *ZmNAC080308* genomic sequence and about 1.5 kb upstream before start site as its promoter region from each of the 199 NLs by PCR using Prime STAR GXL DNA Polymerase (Takara, China) and four pairs of overlapping primers (Supplementary Table 1). The amplified gene fragments were sequenced and assembled by Tsingke Biological Technology (China).

Multiple alignments of DNA sequences and amino acid sequences were performed separately using DNAMAN and MEGA 7.0 software [37]. SNPs and InDels with minor allele frequency greater than 0.05 were identified visually. Phylogenetic analysis of ZmNAC080308 with other NAC proteins was performed by constructing a tree in MEGA 7.0 using the neighbor-joining (NJ) method with a bootstrap of 1000 replications. Evolutionary distances were computed using the p-distance method and all ambiguous positions were removed from each sequence pair [38, 39].

### Linkage disequilibrium (LD) and association analysis of ***ZmNAC080308***

After identifying variants in the *ZmNAC080308* gene, LD analysis was performed using Haploview software [40]. Candidate gene association analysis was conducted using a Mixed Linear Model in Tassel 5.0 [41]. Population structure (Q) and kinship (K) data from previous studies [33, 42] were used to improve the fidelity of GY-associated SNP detection [43]. Markers with *p*-value < 0.05 (-log_10_(*p*) = 1.30) were considered to be significantly associated with GY.

### Subcellular localization of the ZmNAC080308 protein

The vector pRTL2 was digested with the restriction enzymes *Sac*I and *Kpn*I (NEB, USA) for > 3 h. The coding region of *ZmNAC080308* was cloned from Qi319, an NL inbred line, without the terminator codon (TGA) by PCR to include the corresponding 15-bp extensions homologous to the vector ends (sense: 5’-CCGGGAATTCCATGG-3’; antisense: 5’-CTCTAGAATGGTGAG-3’). The digested vector and the PCR product were recovered using a Zymoclean™ Gel DNA Recovery Kit (Zymo Research, USA). We then constructed the recombinant plasmid using an In-Fusion HD Cloning Kit (Takara, China) according to the manufacturer’s instructions. The recombinant plasmid and the empty vector plasmid were transformed into maize leaf protoplasts using the PEG method [44]. After a 16-h incubation in darkness, GFP fluorescence was observed using an LSM880 fluorescence microscope (Zeiss, Germany).

### Transactivation assay

To analyze whether *ZmNAC080308* possessed transactivation activity, the yeast strain AH109 containing *HIS3* and *lacZ* reporter genes was used to express the *ZmNAC080308* gene fused to the GAL4 DNA-binding domain. The full-length coding sequence of *ZmNAC080308* was obtained by PCR using primers: 5’-ATGGCCATGGAGGCCGAATTCATGGCAATGGTGGCGGCG-3’ and 5’-ATGCGGCCGCTGCAGGTCGACTCAGAACGGAGGCAAGAT-3’. The resulting PCR product was cloned downstream of the GAL4 binding domain in the pGBTK7 bait vector digested with *Sal*I (NEB, USA). The recombinant plasmid was then transformed into yeast strain AH109 using Matchmaker™ Gold Yeast Two-Hybrid System (Takara, China) according to the manufacturer’s protocol, the empty vector was separately transformed into AH109 as a negative control. The transformed strains were diluted 1:10^0^, 1:10^1^, 1:10^2^, 1:10^3^. The dilutions were plated on SD/-Trp and SD/-Trp/-His (x-gal) solid medium, respectively.

To compare the transcriptional activation abilities of the the two *ZmNAC080308* haplotypes at the 5’-UTR region, the Dual-Luciferase Reporter Assay System (Promega, USA) was applied. The maize ubiquitin (*Ubi*) promoter and the two *ZmNAC080308* 5’-UTR haplotypes were inserted into the Dual-Luciferase Reporter vector pGreen II 0800, this vector contained a *Renilla* luciferase (REN) gene under the constitutive *CaMV 35 S* promoter as a control (Supplemental Fig. S1C). The three different recombinant plasmids were co-transformed into maize protoplasts separated using a PEG-mediated method as mentioned above. For ABA treatments, the transfected protoplasts were first incubated in solution without ABA [45]. After a 16-h incubation, LUC (luciferase) and REN activities were measured using a fluorescence microscope (Zeiss, Germany).

### Transgenic ***Arabidopsis*** plants construction and stress treatment

The coding region of *ZmNAC080308* was amplified from the maize inbred line of Tie7922 by PCR. The PCR products were then inserted into the pCAMBIA1303 vector under the *CaMV* 35 S promoter. The reconstructed vector was introduced into *Agrobacterium tumefaciens* train after sequencing, and then it was transferred into *Arabidopsis* by floral dip method. The T0 generation seeds of transgenic plants were screened on 1/2 Murashige and Skoog (MS) medium with kanamycin. Then the gained positive plants were transplanted to pots filled with a 2:1 mixture of vermiculite and nutrition soil.

For the drought tolerance assay, the T2 plants were transferred from 1/2 MS medium into pots filled with a 2:1 mixture of vermiculite and nutrition soil at the two-leaves stage. After three weeks of recovery and growth under the well-watered condition, the plants were started to be treated under drought stress by withholding watering for three weeks. Then the plants were recovered by rehydration. Three days later, the survival rate of the plants was recorded.

## Supplementary Information

**Additional file 1.**

**Additional file 2.**

## Data Availability

The datasets used and/or analyzed during the current study are available from the corresponding author upon reasonable request.

## Abbreviations

MAS: Marker-assisted selection
NAC: NAM, ATAF, and CUC
SNAC: Stress related NAC
GY: Grain yield
SNP: Single nucleotide polymorphism
UTR: Untranslated region
TF: Transcription factor
ERSE: Endoplasmic reticulum stress response element
DREB: Dehydration-responsive element-binding
NLs: Chinese inbred lines
ALs: US off-PVP lines
qRT-PCR: Quantitative reverse transcription PCR
ABA: Abscisic acid
MAF: Minor allele frequency
NJ: Neighbor-joining
MLM: Mixed linear model
Ubi: Ubiquitin
LUC: Firefly luciferase
REN: *Renilla* luciferase
GFP: Green fluorescent protein
CDS: Coding DNA sequence
LD: Linkage disequilibrium
WW: Well-watered
WS: Water stressed
HN: Hainan
XJ: Xinjiang

## References

[1] Singh KB, Foley RC, Onate-Sanchez L (2002). Transcription factors in plant defense and stress responses. Current opinion in plant biology.

[2] Souer E, Houwelingen AV, Kloos D, Mol J, Koes R (1996). The No Apical Meristem Gene of Petunia Is Required for Pattern Formation in Embryos and Flowers and Is Expressed at Meristem and Primordia Boundaries. Cell.

[3] Aida M, Ishida T, Fukaki H, Fujisawa H, Tasaka M (1997). Genes involved in organ separation in Arabidopsis: an analysis of the cup-shaped cotyledon mutant. Plant Cell.

[4] Ooka H, Satoh K, Doi K, Nagata T, Otomo Y, Murakami K, Matsubara K, Osato N, Kawai J, Carninci P (2003). Comprehensive analysis of NAC family genes in Oryza sativa and Arabidopsis thaliana. DNA Res.

[5] Nuruzzaman M, Manimekalai R, Sharoni AM, Satoh K, Kondoh H, Ooka H, Kikuchi S (2010). Genome-wide analysis of NAC transcription factor family in rice. Gene.

[6] Li L, Ma YW, Zhang SH, Hao ZF, Li XH (2015). Zea mays NAC transcription factor family members: their genomic characteristics and relationship with drought stress. Res J Biotechnol.

[7] Fang YJ, You J, Xie KB, Xie WB, Xiong LZ (2008). Systematic sequence analysis and identification of tissue-specific or stress-responsive genes of NAC transcription factor family in rice. Mol Genet Genomics.

[8] Nakashima K, Takasaki H, Mizoi J, Shinozaki K, Yamaguchi-Shinozaki K (2012). NAC transcription factors in plant abiotic stress responses. Bba-Gene Regul Mech.

[9] Nuruzzaman M, Sharoni AM, Kikuchi S: Roles of NAC transcription factors in the regulation of biotic and abiotic stress responses in plants. Front Microbiol 2013, 4. 10.3389/fmicb.2013.00248 PMC3759801 24058359

[10] Shao HB, Wang HY, Tang XL: NAC transcription factors in plant multiple abiotic stress responses: progress and prospects. Front Plant Sci 2015, 6. 10.3389/fpls.2015.00902 PMC4625045 26579152

[11] Wang ZY, Dane F (2013). NAC (NAM/ATAF/CUC) transcription factors in different stresses and their signaling pathway. Acta Physiol Plant.

[12] Mao HD, Yu LJ, Han R, Li ZJ, Liu H (2016). ZmNAC55, a maize stress-responsive NAC transcription factor, confers drought resistance in transgenic Arabidopsis. Plant Physiol Bioch.

[13] Mao XG, Zhang HY, Qian XY, Li A, Zhao GY, Jing RL (2012). TaNAC2, a NAC-type wheat transcription factor conferring enhanced multiple abiotic stress tolerances in Arabidopsis. J Exp Bot.

[14] Jeong JS, Kim YS, Baek KH, Jung H, Ha SH, Do Choi Y, Kim M, Reuzeau C, Kim JK (2010). Root-Specific Expression of OsNAC10 Improves Drought Tolerance and Grain Yield in Rice under Field Drought Conditions. Plant physiology.

[15] Hu HH, Dai MQ, Yao JL, Xiao BZ, Li XH, Zhang QF, Xiong LZ (2006). Overexpressing a NAM, ATAF, and CUC (NAC) transcription factor enhances drought resistance and salt tolerance in rice. P Natl Acad Sci USA.

[16] Zheng XN, Chen B, Lu GJ, Han B (2009). Overexpression of a NAC transcription factor enhances rice drought and salt tolerance. Biochem Bioph Res Co.

[17] Hu HH, You J, Fang YJ, Zhu XY, Qi ZY, Xiong LZ: Characterization of transcription factor gene SNAC2 conferring cold and salt tolerance in rice (vol 67, pg 169, 2008). Plant Molecular Biology 2010, 72(4–5):567–568. 10.1007/s11103-008-9309-5 18273684

[18] Jeong JS, Kim YS, Redillas MCFR, Jang G, Jung H, Bang SW, Choi YD, Ha SH, Reuzeau C, Kim JK (2013). OsNAC5 overexpression enlarges root diameter in rice plants leading to enhanced drought tolerance and increased grain yield in the field. Plant Biotechnol J.

[19] Nakashima K, Tran LSP, Van Nguyen D, Fujita M, Maruyama K, Todaka D, Ito Y, Hayashi N, Shinozaki K, Yamaguchi-Shinozaki K (2007). Functional analysis of a NAC-type transcription factor OsNAC6 involved in abiotic and biotic stress-responsive gene expression in rice. Plant J.

[20] Chen X, Wang YF, Lv B, Li J, Luo LQ, Lu SC, Zhang X, Ma H, Ming F (2014). The NAC Family Transcription Factor OsNAP Confers Abiotic Stress Response Through the ABA Pathway. Plant Cell Physiol.

[21] Mao HD, Wang HW, Liu SX, Li Z, Yang XH, Yan JB, Li JS, Tran LSP, Qin F: A transposable element in a NAC gene is associated with drought tolerance in maize seedlings. Nat Commun 2015, 6. 10.1038/ncomms9326 PMC4595727 26387805

[22] Huang QJ, Wang Y, Li B, Chang JL, Chen MJ, Li KX, Yang GX, He GY: TaNAC29, a NAC transcription factor from wheat, enhances salt and drought tolerance in transgenic Arabidopsis. BMC plant biology 2015, 15. 10.1186/s12870-015-0644-9 PMC4632686 26536863

[23] Waters AJ, Makarevitch I, Noshay J, Burghardt LT, Hirsch CN, Hirsch CD, Springer NM (2017). Natural variation for gene expression responses to abiotic stress in maize. Plant J.

[24] Wang XL, Wang HW, Liu SX, Ferjani A, Li JS, Yan JB, Yang XH, Qin F (2016). Genetic variation in ZmVPP1 contributes to drought tolerance in maize seedlings. Nat Genet.

[25] Xiang YL, Sun XP, Gao S, Qin F, Dai MQ (2017). Deletion of an Endoplasmic Reticulum Stress Response Element in a ZmPP2C-A Gene Facilitates Drought Tolerance of Maize Seedlings. Mol Plant.

[26] Liu SX, Wang XL, Wang HW, Xin HB, Yang XH, Yan JB, Li JS, Tran LSP, Shinozaki K, Yamaguchi-Shinozaki K et al: Genome-Wide Analysis of ZmDREB Genes and Their Association with Natural Variation in Drought Tolerance at Seedling Stage of Zea mays L. Plos Genet 2013, 9(9). 10.1371/journal.pgen.1003790 PMC3784558 24086146

[27] Lu M, Ying S, Zhang DF, Shi YS, Song YC, Wang TY, Li Y (2012). A maize stress-responsive NAC transcription factor, ZmSNAC1, confers enhanced tolerance to dehydration in transgenic Arabidopsis. Plant cell reports.

[28] Flint-Garcia SA, Thornsberry JM, Buckler ES (2003). Structure of linkage disequilibrium in plants. Annu Rev Plant Biol.

[29] Thornsberry JM, Goodman MM, Doebley J, Kresovich S, Nielsen D, Buckler ES (2001). Dwarf8 polymorphisms associate with variation in flowering time. Nat Genet.

[30] Lopez-Ochoa L, Acevedo-Hernandez G, Martinez-Hernandez A, Arguello-Astorga G, Herrera-Estrella L (2007). Structural relationships between diverse cis-acting elements are critical for the functional properties of a rbcS minimal light regulatory unit. J Exp Bot.

[31] Shariatipour N, Heidari B: Investigation of Drought and Salinity Tolerance Related Genes and their Regulatory Mechanisms in Arabidopsis (Arabidopsis thaliana). The Open Bioinformatics Journal 2018, 11(1).

[32] Hao ZF, Li XH, Su ZJ, Xie CX, Li MS, Liang XL, Weng JF, Zhang DG, Li L, Zhang SH (2011). A proposed selection criterion for drought resistance across multiple environments in maize. Breeding Sci.

[33] Wang N, Wang ZP, Liang XL, Weng JF, Lv XL, Zhang DG, Yang J, Yong HJ, Li MS, Li FH (2016). Identification of loci contributing to maize drought tolerance in a genome-wide association study. Euphytica.

[34] Wang N, Li L, Gao W-w, Wu Y, Yong H, Weng J, Li M, Zhang D, Hao Z, Li X (2017). Transcriptomes of early developing tassels under drought stress reveal differential expression of genes related to drought tolerance in maize. Journal of Integrative Agriculture.

[35] Livak KJ, Schmittgen TD (2001). Analysis of relative gene expression data using real-time quantitative PCR and the 2(T)(-Delta Delta C) method. Methods.

[36] Lescot M, Dehais P, Thijs G, Marchal K, Moreau Y, Van de Peer Y, Rouze P, Rombauts S (2002). PlantCARE, a database of plant cis-acting regulatory elements and a portal to tools for in silico analysis of promoter sequences. Nucleic acids research.

[37] Kumar S, Stecher G, Tamura K: MEGA7: Molecular Evolutionary Genetics Analysis Version 7.0 for Bigger Datasets. Molecular biology and evolution 2016, 33(7):1870–1874. 10.1093/molbev/msw054 PMC8210823 27004904

[38] Felsenstein J (1985). Confidence Limits on Phylogenies: an Approach Using the Bootstrap. Evolution.

[39] Saitou N, Nei M (1987). The neighbor-joining method: a new method for reconstructing phylogenetic trees. Molecular biology and evolution.

[40] Barrett JC, Fry B, Maller J, Daly MJ (2005). Haploview: analysis and visualization of LD and haplotype maps. Bioinformatics.

[41] Bradbury PJ, Zhang Z, Kroon DE, Casstevens TM, Ramdoss Y, Buckler ES (2007). TASSEL: software for association mapping of complex traits in diverse samples. Bioinformatics.

[42] Liu CL, Hao ZF, Zhang DG, Xie CX, Li MS, Zhang XC, Yong HJ, Zhang SH, Weng JF, Li XH: Genetic properties of 240 maize inbred lines and identity-by-descent segments revealed by high-density SNP markers. Molecular Breeding 2015, 35(7).

[43] Yu JM, Pressoir G, Briggs WH, Bi IV, Yamasaki M, Doebley JF, McMullen MD, Gaut BS, Nielsen DM, Holland JB (2006). A unified mixed-model method for association mapping that accounts for multiple levels of relatedness. Nat Genet.

[44] Cho HY, Lee C, Hwang SG, Park YC, Lim HL, Jang CS (2014). Overexpression of the OsChI1 gene, encoding a putative laccase precursor, increases tolerance to drought and salinity stress in transgenic Arabidopsis. Gene.

[45] Kim N, Moon SJ, Min MK, Choi EH, Kim JA, Koh EY, Yoon I, Byun MO, Yoo SD, Kim BG: Functional characterization and reconstitution of ABA signaling components using transient gene expression in rice protoplasts. Front Plant Sci 2015, 6. 10.3389/fpls.2015.00614 PMC4524894 26300907

