# Supplementary material for: Natural variations in the non-coding region of ZmNAC080308 contributes maintaining grain yield under drought stress in maize
Source: BMC Plant Biol. 2021 Jun 30;21:305. doi: 10.1186/s12870-021-03072-9 (PMC8243440; doi:10.1186/s12870-021-03072-9)
Supplement: Supplementary file 1 — Additional file 1. [file 12870_2021_3072_MOESM1_ESM.docx]

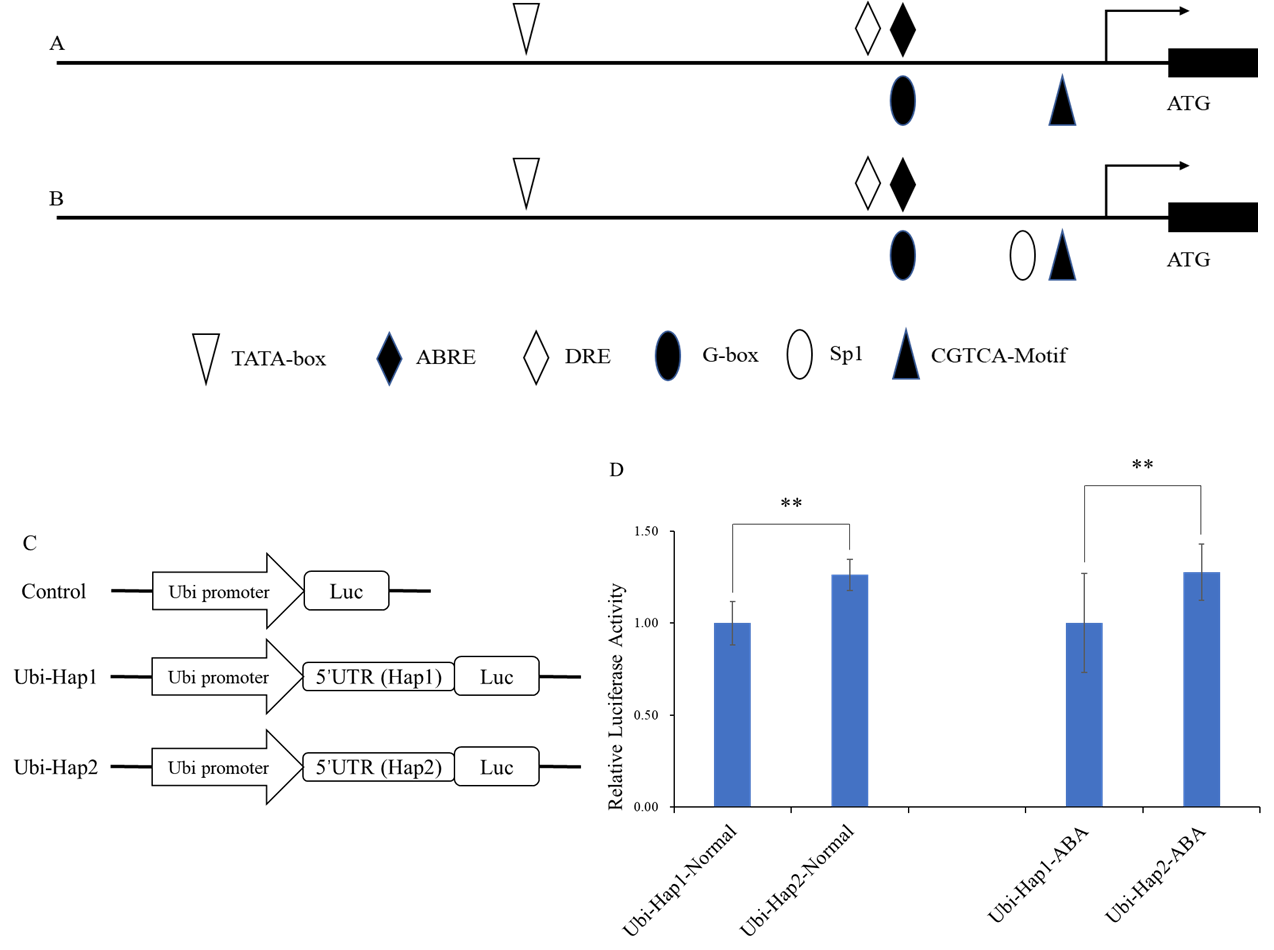


Supplemental Figure S1 Distribution of *cis*-acting regulatory elements and transcriptional activation of 5’-UTR of *ZmNAC080308*. (A) Distribution of several *cis*-elements in Hap1 genotypes and (B) Hap2 genotypes; (C) Vector construction for the Dual-Luciferase Reporter Assay system; (D) The LUC to REN ratio controlled by the *Ubiquitin* (*Ubi*) promoter or the 5’-UTR of the two *ZmNAC080308* haplotypes. Each data point is the mean (± SD) of 12 replicates (Student’s *t*-test).

Supplemental Table 1 Primers used for amplification of *ZmNAC080308* genomic sequence

| Primers | Sequence | Amplified region |
| --- | --- | --- |
| Q1-F | GTCGGGAAAAGCTCAGTT | Promoter |
| Q1-R | ATAGAATCGCGTTGTCCG |  |
| 5U-F | GCACGAGCTGGGCTACGC | 5’ UTR |
| 5U-R | GATGATGGGCACGGGCAG |  |
| SNAC8308-F | GGACAACGCGATTCTATTTATT | ATG-TGA |
| SNAC8308-R | CCTTACAAGACTACCAGCAACA |  |
| 3U-F | CGACGAAGATGTGGCAGAC | 3’ UTR |
| 3U-R | GACCAATCTCGGTTCAGG |  |
