# Supplementary material for: Natural variations in the non-coding region of ZmNAC080308 contributes maintaining grain yield under drought stress in maize
Source: BMC Plant Biol. 2021 Jun 30;21:305. doi: 10.1186/s12870-021-03072-9 (PMC8243440; doi:10.1186/s12870-021-03072-9)
Supplement: Supplementary file 2 — Additional file 2. [file 12870_2021_3072_MOESM2_ESM.docx]

**Natural variations in the non-coding region of *ZmNAC080308* contributes to maintain grain yield under drought stress in maize**

Nan Wang^1,3,4,5#^, Ming Cheng^1,2,#^, Yong Chen^1^ , Bojuan Liu^1^, Xiaonan Wang^1^,Guojun Li^1^, Yueheng Zhou^1^, Ping Luo^1^, Zhangying Xi^2^, Hongjun Yong^1^, Degui Zhang^1^, Mingshun Li^1^, Xuecai Zhang^3^, Felix San Vicente^3^, Zhuanfang Hao^1*^, Xinhai Li^1*^

^1^Institute of Crop Sciences, Chinese Academy of Agricultural Sciences, Beijing, P.R. China

^2^College of Agronomy, Henan Agricultural University, Zhengzhou, P.R. China

^3^International Maize and Wheat Improvement Center (CIMMYT), Texcoco, Mexico

^4^College of Agronomy, Hebei Agricultural University, Baoding, P.R. China

^5^North China Key Laboratory for Crop Germplasm Resources of the Education Ministry, Hebei Agricultural University, Baoding, P.R. China

^#^These authors have equal contribution to this paper.

^*^Corresponding authors

Dr. Zhuanfang Hao, haozhuanfang@163.com;

Dr. Xinhai Li, lixinhai@caas.cn.


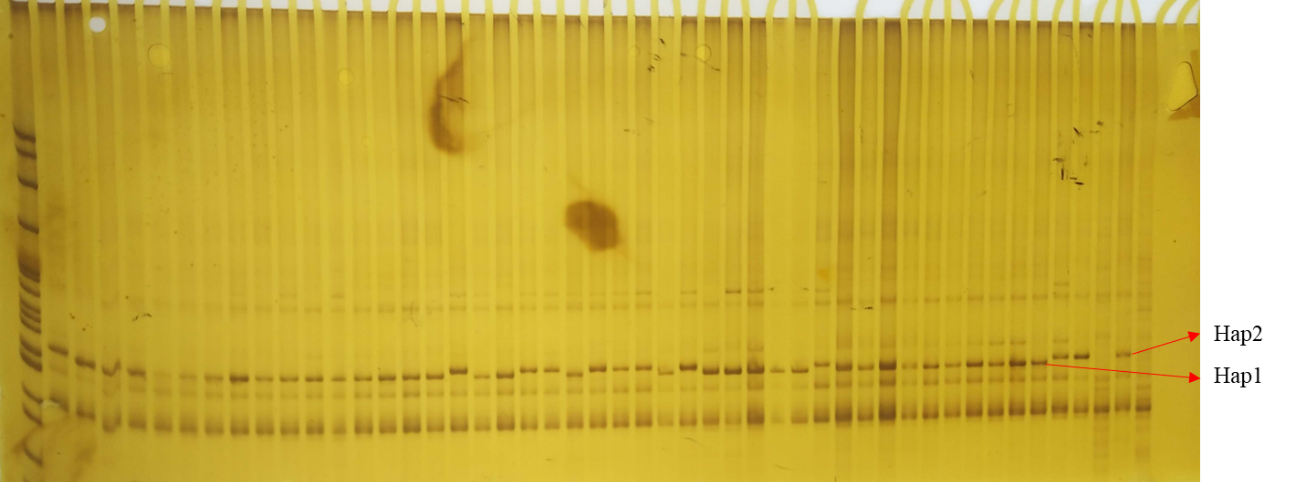


Supplementary Figure S2 Two genotypes in the off-PVP panel were identified using the functional marker developed based on the two InDels (Marker 80 and Marker 81) in the 5’ UTR of ZmNAC080308.
